# A Purified Platelet-Derived Exosome Product for Chronic Wound Healing: A Novel Therapeutic Strategy and Next-Generation Delivery Platform

**DOI:** 10.3390/pharmaceutics18020222

**Published:** 2026-02-09

**Authors:** Rou Wan, Ken Nishimura, Atta Behfar, Chunfeng Zhao, Steven L. Moran

**Affiliations:** 1 Division of Plastic Surgery, Mayo Clinic, Rochester, MN 55905, USA; wan.rou@mayo.edu (R.W.);; 2 Department of Cardiovascular Medicine, Mayo Clinic, Rochester, MN 55905, USA; 3 Van Cleve Cardiac Regenerative Medicine Program, Center for Regenerative Medicine, Mayo Clinic, Rochester, MN 55905, USA; 4 Department of Orthopedic Surgery, Mayo Clinic, Rochester, MN 55905, USA

**Keywords:** exosome, wound healing, drug delivery, clinical translation

## Abstract

Chronic wounds remain a major unmet clinical challenge, often failing to progress to normal healing due to persistent inflammation, impaired angiogenesis, and cellular senescence. Exosomes have recently been investigated as promising acellular therapeutics capable of restoring intercellular communication and promoting tissue regeneration. Among these, the Purified Exosome Product (PEP) represents a next-generation, platelet-derived exosome formulation manufactured under Good Manufacturing Practice (GMP) conditions with high purity, stability, and reproducibility. This review summarizes the current advances in exosome-based chronic wound therapeutics and PEP delivery systems and their translational potentials. Incorporation of PEP into bioengineered carriers such as fibrin sealant, collagen scaffolds, and hyaluronic acid (HA) hydrogels enables localized and sustained exosome release, significantly prolonging therapeutic effects and improving regenerative outcomes. Fibrin-based PEP delivery achieved complete wound closure and functional skin regeneration in animal models, while collagen and HA-based systems showed promising results for injectable and dermatologic applications. Beyond its intrinsic healing effects, PEP may also serve as a nanocarrier for other drugs, offering a future direction in chronic wound management.

## 1. Introduction

Chronic wounds represent a major global health and economic burden, affecting millions of people with diabetes, vascular disease, radiation injury, and other comorbidities [1]. These wounds fail to progress through the normal stages of healing, resulting in persistent inflammation, impaired angiogenesis, fibroblast senescence, and extracellular matrix (ECM) disorganization [2,3]. Despite progress in advanced dressings, bioengineered skin substitutes, and growth factor therapies, most current treatments for chronic wounds remain largely supportive and rarely restore the native skin architecture and function. In fact, many of the current treatments focus on symptom management, such as maintaining moisture balance, controlling infection, or providing temporary coverage, rather than actively regenerating functional skin tissues [1,2,3]. Even though growth factors have shown preliminary improvements in tissue regeneration, their effects are often transient due to rapid degradation within the wound microenvironment. Moreover, the delivery of bioactive molecules to the wound bed is challenged by enzymatic breakdown, short half-life, and poor tissue penetration [3,4,5]. Regenerative therapeutics are often paused at the preclinical level due to limited improvements seen during clinical translation [6].

**Figure 1:**
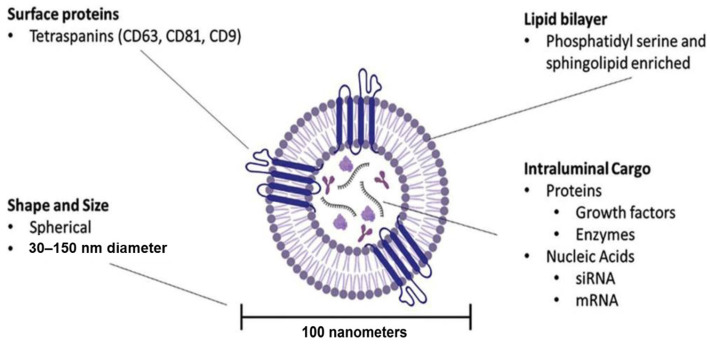
Overview of exosome architecture and components. Biomolecular cargo (e.g., proteins and nucleic acids) is selectively loaded into the intraluminal space for extracellular trafficking. (Reprinted from Ref. [10]).

The emergence of biological therapies and drug delivery systems for chronic wounds offers new opportunities for more effective and regenerative healing outcomes. Among these, extracellular vesicles (EVs), particularly exosomes, have gained the spotlight recently as natural nanocarriers capable of delivering functional proteins, nucleic acids, and lipids directly to target cells [7,8,9]. Exosomes are small particles, typically 30–150 nm in diameter, which are secreted by almost all cell types in the human body and play critical roles in intercellular communication. They originate from multivesicular bodies of the endosomal pathway and are released into the extracellular space through exocytosis. Their lipid bilayer protects their cargo from degradation and enables efficient uptake by recipient cells (Figure 1) [7,8,9,10]. However, translating cell-derived exosome strategies into clinical use remains challenging due to their donor variability, tricky commercial production process, varying purity, and limited storage stability. These barriers have driven the development of next-generation exosome products, such as Purified Exosome Product (PEP), which aims to integrate biological efficacy with clinical-grade quality and commercial-scale reproducibility [6,9,10]. This review will summarize advances in exosome-based systems for chronic wound management, with a focus on platelet-derived PEP, highlighting its delivery strategies, and translational potential.

## 2. Exosomes in Chronic Wound Healing

Physiologically, exosomes transport bioactive molecules between cells which then regulate numerous physiological and pathological processes [7,9,11,12,13,14,15,16]. In chronic wounds, where normal healing and cellular communication are disrupted, exosome can restore cellular crosstalk and activate important regenerative pathways. These include PI3K/Akt, ERK1/2, Wnt/β-catenin, TGF-β/Smad, and VEGF/HIF-1α signaling, which contribute to various phases of wound healing [9,10,11,12,17,18,19,20,21,22,23].

### 2.1. Current Role and Advances of Exosomes in Chronic Wound Management

Exosomes offer a cell-free alternative in wound management that overcomes many of the challenges associated with stem cell therapy, such as issues of cell survival, immune rejection, high costs and complex regulatory processes, while preserving the regenerative and immunomodulatory properties of their parent cells [9,13,21,24]. Numerous preclinical studies have shown exosomes to effectively restore normal wound healing in difficult-to-treat wounds (e.g., diabetic wounds, ischemic wounds) by:

- **Promoting angiogenesis** through upregulating proangiogenic factors such as VEGF, PDGF, and angiopoietin-1 [17,25,26,27,28];
- **Modulating inflammation** through balancing pro- and anti-inflammatory cytokines, reducing macrophage M1 polarization, and promoting M2 phenotypes [17,28,29,30];
- **Stimulation of ECM remodeling and re-epithelialization** through stimulating the proliferation and migration of keratinocytes and fibroblasts [17,27,28,31];
- **Improve tissue energy homeostasis and cell survival** through enhancing cellular metabolism and mitochondrial transfer [31,32].

In order to enhance the therapeutic durability, concentration and targeted delivery of exosomes, a variety of bioengineering technologies and delivery systems have been investigated. These have included loading exosomes into hydrogels, scaffolds, patches, and aerosol formulations to enable localized and sustained release to wound sites [24,33,34,35,36,37]. These carriers protect exosomes from degradation or clearance, prolong their biological activities, and enable controlled delivery to target tissues. For example, Wang et al. encapsulated bioactive adipose-derived exosomes within a polypeptide-based hydrogel, which contained Pluronic F127, oxidative HA, and Poly-ε-L-lysine. This successful encapsulation allowed sustained pH-responsive release of exosomes at the wound site, while also providing antibacterial capacity, necessary mechanical properties, better biocompatibility and injectability [34]. Similarly, Zhao et al. evaluated HA as a delivery system for exosomes and performed successful intra-articular injection using a 25-gauge needle under ultrasound guidance. And they achieved daily release of PEP particles from the HA matrix for up to 7 days [38]. Encapsulation within hydrogel media can significantly improve exosome retention, enhancing therapeutic effects of exosomes in full-thickness wounds.

Among the various sources of exosomes, mesenchymal stem cell-derived exosomes (MSC-Exos) have been extensively studied due to their regenerative potential. MSC-Exos consistently accelerate wound closure, enhance angiogenesis, modulate inflammation, and promote re-epithelialization and collagen deposition in cellular and animal models of chronic wounds, including diabetic ulcers and burns [9,16,21,39].

In comparison, platelet-derived exosomes (pExos) have been less studied but are now recognized as a promising alternative to cell derived exosomes owing to their high accessibility, lower cost, and favorable safety profile compared to MSC-Exos [9,12,18,40,41]. pExos can be readily obtained from human platelets, which are abundant and easily collected through routine blood draws or platelet-rich plasma (PRP). They are also naturally enriched with a broad spectrum of pro-angiogenic and pro-hemostatic growth factors, such as PDGF, VEGF, and TGF-β, which are essential for tissue repair and angiogenesis. Furthermore, because platelets are already FDA-approved for transfusion, their use as an exosome source offers a clearer regulatory and translational advantage over stem cell-based therapies [17,18,42,43].

Beyond their intrinsic regenerative capacity, exosomes are being actively explored as biological nanocarriers for therapeutic delivery. They are capable of transporting drugs, peptides, genetic materials and growth factors to target tissues, further expanding their potential applications in regenerative medicine and wound healing [24,44,45,46,47]. When used as delivery vehicles, exosomes have shown synergistic effects on angiogenesis, collagen deposition, and epithelial repair in chronic wound models. For example, exosomes engineered to carry miR-21 have been shown to enhance keratinocyte migration and re-epithelialization [48]. Similarly, engineered exosomes loaded with miR-31 significantly promoted diabetic wound healing by enhancing angiogenesis, fibrogenesis, and re-epithelization [49].

### 2.2. Current Challenges in the Clinical Translation of Exosome in Wound Healing

While holding great promises for chronic wound healing, exosomes face several limitations that need to be addressed for successful clinical translation. The main challenges include the lack of standardized and scalable methods for exosome isolation and purification, which often result in low yields and contamination with other extracellular vesicles. Conventional isolation techniques such as ultracentrifugation are labor-intensive and may damage the delicate structure of exosomes, reducing their bioactivity and clinical utility [9,23,46,50,51,52,53]. Therefore, achieving large-scale, high-purity production suitable for therapeutic use remains a major concern for exosome clinical applications. Furthermore, as a drug delivery system, achieving consistent and high drug-loading efficiency remains a challenge compared to other nanocarriers such as liposomes and polymeric nanoparticles (PLGA) [8,51,54].

Another critical aspect is exosome stability, preservation, and transportation. Exosomes possess an inherently short half-life and a tendency to aggregate with cells and other particles, making long-term storage and targeted delivery to the wound site challenging [55,56]. In vivo, exosomes can face rapid clearance by the reticuloendothelial system, limiting their circulation time and targeted accumulation [24,46,53,55]. While lyophilization has shown benefits in preservation, standardized protocols and optimized cryoprotectants are still required to maintain exosome stability and bioactivity. Moreover, the high cost of current exosome preparations and storage also limits the feasibility of widespread clinical use [24,53,54,55,56,57].

Addressing these limitations through innovations in exosome isolation, purification, bioengineering, targeted delivery technologies, and the establishment of affordable processes is crucial for translating exosome-based therapies into effective treatments for chronic wounds to benefit millions of patients.

## 3. Clinically Available Exosomes (Purified Exosome Product)

**Figure 2:**
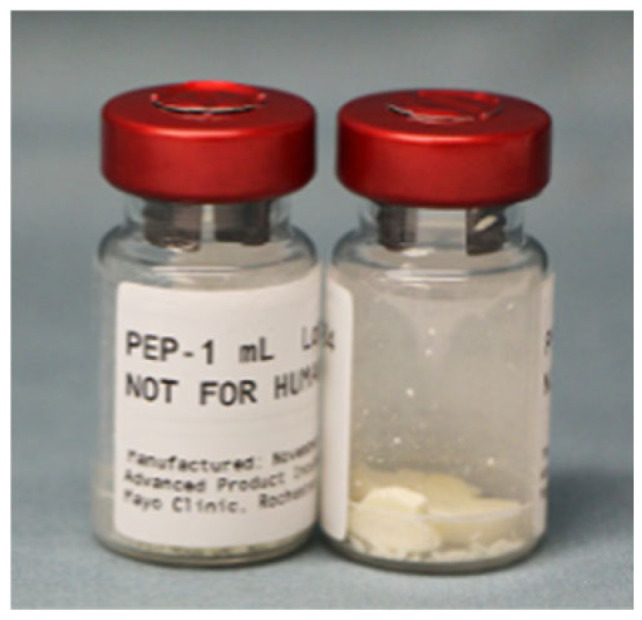
The PEP is supplied as a lyophilized powder sealed in a glass vial and stored at room temperature.

Purified Exosome Product (PEP), which is a clinical-grade, lyophilized exosome powder derived from human platelets has been introduced in Phase I and Phase II FDA trials. It is derived from platelets in donated human blood, a source with an established history of FDA compatibility and a reliable supply. This origin aligns well with existing regulatory frameworks for blood-derived biologics. Each vial of PEP contains up to 2 trillion bioactive exosomes, equivalent to approximately 200 billion exosomes per milliliter, and is shelf-stable for up to 24 months at room temperature (Figure 2) [17,58,59]. PEP is manufactured under Good Manufacturing Practice (GMP) conditions using a patented process that ensures high-purity isolation, consistent particle size distribution, and preserved bioactivity. Each batch undergoes rigorous characterization for exosome size, surface markers (CD63, CD81), and functional bioactivity. PEP is one of the few exosome therapeutics ready for large-scale clinical application [59,60,61,62]. PEP can be produced at large scale due to its derivation from donated human platelets supported by existing blood-bank infrastructure. In addition, the patented manufacturing process employs multiple staged operations of ultrafiltration and purification to remove cellular debris and impurities, resulting in a pure and consistent exosome population in the final sterile product (US Patent 20160324A1) [59,63]. This cell-free, GMP-compatible process contrasts with many other experimental exosomes, which rely on prolonged cell culture, donor variability, and pose challenges for large-scale production. PEP in this review was produced by Rion, Inc. (Rochester, MN, USA), in collaboration with the Mayo Clinic Center for Regenerative Medicine. Each lot of PEP undergoes comprehensive quality control testing to ensure purity, potency, safety, and lot-to-lot consistency [50,60].

At present, PEP represents the only clinical-grade exosome therapeutic with reported application in wound management in humans. While multiple exosome-based products derived from other sources are undergoing clinical evaluation for a range of indications, none have been specifically developed for wound healing. PEP has demonstrated promising outcomes in healing chronic wounds. It accelerates epithelialization and promotes vascular ingrowth. Currently, PEP is progressing through the standard regulatory pathway for biologics, including an Investigational New Drug (IND) application and clinical trials. Clinical trials are underway to evaluate its safety and efficacy in DFUs (https://clinicaltrials.gov/study/NCT06319287, accessed on 11 November 2025) and chronic radiation ulcers (https://clinicaltrials.gov/study/NCT06793748, accessed on 11 November 2025). In a published case report, PEP was successfully used to treat nonhealing scalp wounds following radiation therapy, showing successful tissue regeneration even in previous treatment-resistant cases [64]. The remaining hurdles for its clinical translation include demonstrating consistent efficacy in difficult-to-heal wounds and establishing standardized dosing regimens across various clinical applications.

PEP-only cannot provide sustainable and controlled release at wound sites. Qi et al. and Shi et al. initially evaluated PEP delivery and cellular uptake in vitro by dissolving PEP directly in phosphate-buffered saline (PBS) [59,65]. Similarly, Miller et al. tested PEP uptake following direct reconstitution in sterile water [66]. These vehicles were primarily employed for in vitro investigations. When administered in solution form, PEP demonstrated rapid cellular internalization and strong bioactivity across multiple cell types, including tenocytes, chondrocytes, stromal cells, and epithelial cells [17,59,65,66,67,68].

PEP mixed in PBS or water exhibited fast cell internalization and effective cell absorption by targeted cells in vitro due to its strong ability to interact with cell membranes, as confirmed by fluorescently labeled PEP [59,65]. While aqueous PEP is excellent for cell-based studies, it is not designed for in vivo wound delivery. The rapid cell uptake and short half-life result in potent but transient and unstable therapeutic effects [17,65,66]. Therefore, slow-release carriers are required for chronic wound applications in vivo [17]. We have evaluated various sustained-release scaffolds and hydrogels for PEP release in vitro, such as HA-based gel and fibrin glue for cell studies. But these thicker carriers interfered with cell culture conditions and microscopic visualization in vitro, leading to failure of experiments.

PEP has also been administered in vivo in aqueous form (water or PBS) for systemic or inhaled delivery [60,69]. In a porcine pulmonary biodistribution model, PEP was labeled with fluorescent DiR and delivered via nebulization, intravenous infusion, or pulmonary artery balloon catheter. Nebulization resulted in efficient uptake by airway epithelium and tracheal tissue, while catheter-guided delivery demonstrated the ability to localize exosomes to a single area within the lung. No significant off-target accumulation was observed. Intravenous administration of PEP-PBS achieved successful global delivery [69]. Based on these distribution findings, nebulized PEP-water was subsequently tested in a cigarette-induced emphysema mouse model, resulting in reduced oxidative injury, inflammation, and apoptosis [60]. These results support the feasibility of systemic and inhaled delivery routes for internal organ healing from injuries. In contrast, for cutaneous wounds, reconstituted PEP alone is not recommended, as sustained retention at the wound bed requires a scaffold or carrier. PEP alone is not intended for in vivo or clinical translation, where controlled-release systems are needed to maintain local bioavailability and maximize regenerative outcomes [17,65].

Therefore, a good delivery system is required for such a promising regenerative therapeutic to optimize regenerative outcomes. Sustained release of exosomes is essential to provide a continuous therapeutic effect at the wound site, rather than a short-lived, transient exposure to the bioactive particles.

## 4. Drug Delivery Systems for Purified Exosome Product (PEP)

Investigations have been focused on identifying the optimal clinical-grade delivery carriers for PEP, including fibrin sealant, HA, and collagen scaffolds (Table 1) [9]. Materials that have already been widely used in clinical practice were favored to maximize the potential for clinical translation. Below is a summary of different delivery systems that have been evaluated as PEP carriers.

### 4.1. PEP-Tisseel (Fibrin Sealant)

**Figure 3:**
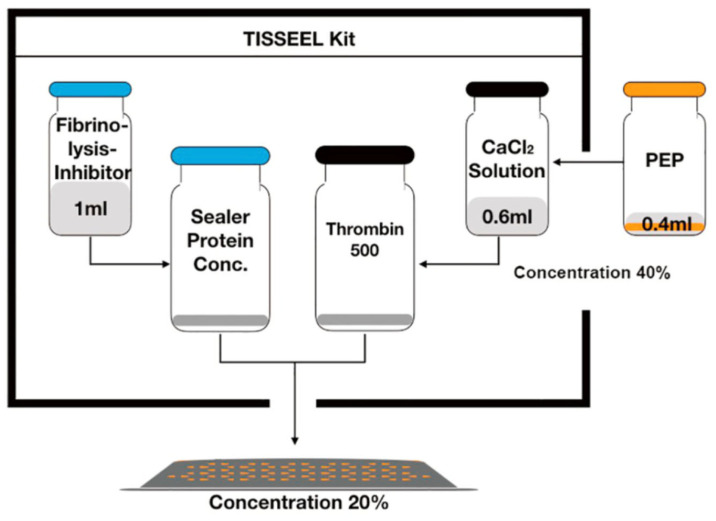
Preparation of Tisseel with PEP (for 20% concentration). Reprinted with permission from Ref. [68] Copyright (2021) (Elsevier).

**Figure 4:**
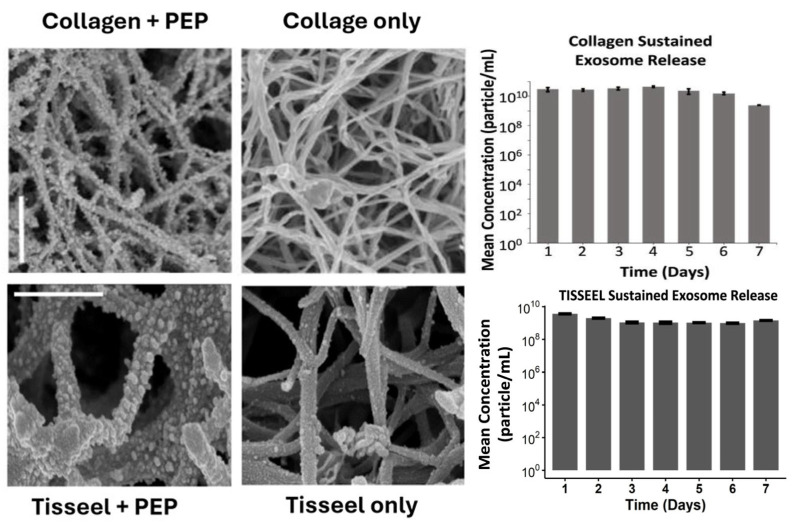
Scanning electron microscopy (SEM) images of Tisseel (scale = 500 nm) and collagen (scale = 1 µm) reconstituted with 1 × 1012 exosomes/mL PEP, showing characteristic “bead-on-a-string” pattern. PEP showed sustained release from both collagen and Tisseel carrier. (Reprinted from Ref. [50]).

**Figure 5:**
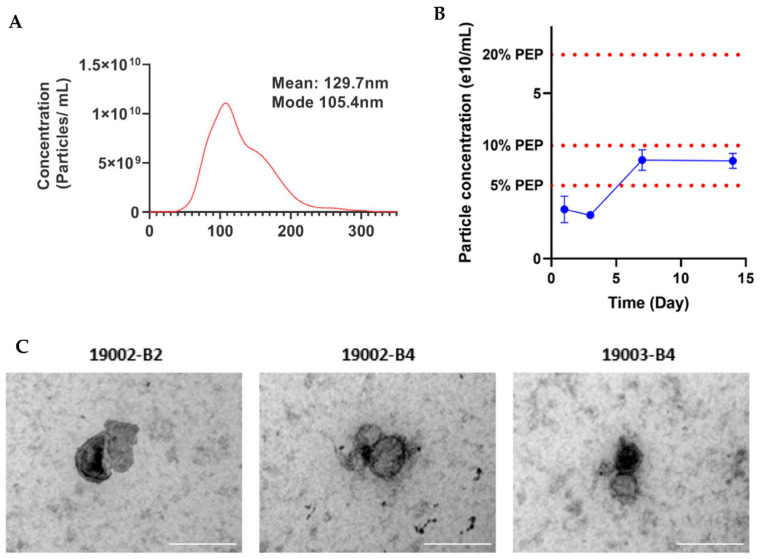
Characterization and release analysis of PEP-Tisseel. (**A**) Size distribution of PEP particles assessed by NanoSight. (**B**) Nanoparticle tracking analysis showing particle concentration of PEP and continuous release from Tisseel fibrin sealant for 14 days. (**C**) Representative images of round and cup-shaped PEP particles by transmission electron microscopy (TEM) from various batches. Scale bar, 200 nm (Reprinted from Ref. [17]).

Tisseel (Baxter, Deerfield, IL, USA) is an FDA-approved, commercially available fibrin sealant composed of human fibrinogen and thrombin that polymerizes in situ to form a stable fibrin hydrogel. It is widely used in clinical practice among surgeons for hemostasis, tissue sealing, and wound closure [82]. Tisseel was considered as a clinically practical delivery vehicle for PEP to support local retention and controlled release in wounds due to its strong tissue adhesion, biocompatibility and clinical readiness (Figure 3). PEP-Tisseel has demonstrated prolonged exosome release for up to 2 weeks and produced a characteristic ultrastructural “bead-on-a-string” pattern along fibrin fibers, demonstrating uniform vesicle attachment and gradual detachment during degradation (Figure 4) [50]. In addition, release assays confirmed particle diameters near 100 nm, consistent with exosomes, and revealed regulated release profiles with ~5–10% sustained PEP release across 14 days (Figure 5) [17,68,70,71].

**Figure 6:**
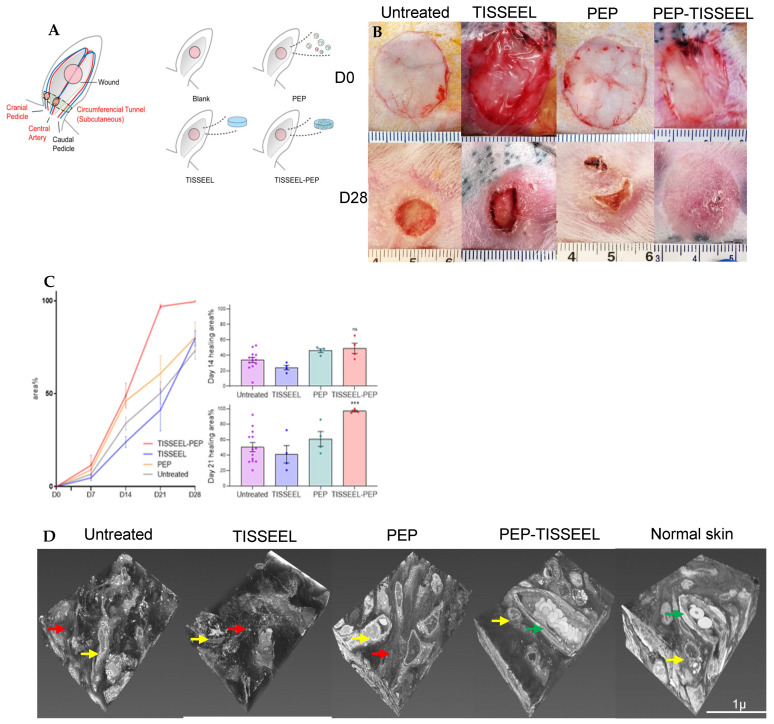
PEP-Tisseel biogel promotes ischemic wound healing in vivo. (**A**) The rabbit ear ischemic wound model and treatment groups (**B**) Ischemic wounds achieved complete closure in the PEP-Tisseel group but not in the PEP-only group. (**C**) Quantification of the healing area confirmed that PEP-only treatment produced early therapeutic effects but failed to sustain healing through day 28. (**D**) 3D-EM reconstruction of wound tissue. Yellow arrow: fibroblast. Red arrow: unorganized collagen deposition. Green arrow: newly formed capillaries with red blood cells. Representative images of 3D-EM projection of skin tissues from each group. (*** *p* < 0.001, ns: not significant) (Reprinted from Ref. [17]).

The efficacy of Tisseel as a carrier for PEP was evaluated in a recent study by Shi et al. [17] PEP-Tisseel biogel was applied to a ischemic rabbit ear model and outcomes were compared to PEP-only treatment in vivo. PEP-Tisseel achieved complete wound closure with functioning regenerated skin by day 28, whereas PEP-only and Tisseel-only groups exhibited persistent wound defects [17]. Their findings confirmed that rapid release in the PEP-only group could only affect early phases of wound repair but was insufficient to sustain continuous effects for 28 days (Figure 6) [17]. Similar benefits were observed in nerve repair, tendon repair and rotator cuff repair [67,68,70,71].

**Figure 7:**
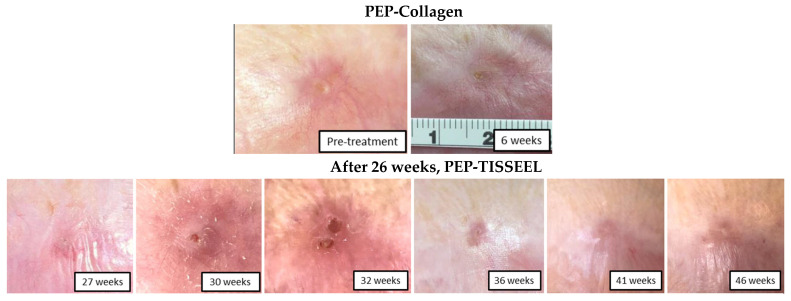
Images of the irradiated scalp wound following Purified Exosome Product (PEP) therapy with complete wound closure at 46 weeks. A collagen (Bellafill [Suneva Medical Inc.])-PEP mixture was initially applied at week 0 and then sealed with a polyurethane dressing (Tegaderm [3M]). PEP-Tisseel treatment began at week 26 due to leakage of PEP under the Tegaderm. Reprinted with permission from Ref. [64] Copyright (2024) (Mayo Foundation for Medical Education and Research).

Ongoing clinical trials are in progress to assess the efficacy of PEP-Tisseel in challenging wounds. Moreover, in a case reported by Pumford et al., an elderly male patient in his 60s developed a chronic nonhealing frontal scalp wound following surgical resection and adjuvant chemoradiation for high-grade angiosarcoma. The wound measured 0.7 cm × 1.1 cm and persisted for 7 months despite extensive conventional wound management, including local wound care, topical antimicrobial therapy, serial debridement, negative pressure wound therapy, and prior surgical reconstruction, all of which failed to achieve wound closure in the irradiated tissue bed [64]. In this case, treatment initially began with a PEP-collagen hydrogel. However, product leakage under the Tegaderm dressing (3M, Maplewood, MN, USA) resulted in reduced therapeutic effects and persistence of the wound. At week 26, the carrier was switched to the thicker Tisseel to improve prevent PEP leakage, which led to successful epithelialization and complete wound closure afterwards (Figure 7) [64]. Currently, we are applying PEP-Tisseel in clinical trials for chronic radiation ulcers and diabetic ulcers as mentioned above. Based on our experience, we believe this delivery method is useful and promising for clinical translation.

### 4.2. PEP-Collagen

For clinical translation, in addition to Tisseel, FDA-approved collagen is another good delivery option for PEP. Rolland et al. analyzed and compared the release profile of PEP-collagen and PEP-Tisseel and found that they were similar as shown in Figure 7 [50]. Both carriers created an ultrastructural “bead-on-a-string” pattern under scanning electron microscopy and achieved sustained exosome release (Figure 5) [50,81].

Since their microscopic appearance and release profiles are comparable, the choice between collagen and Tisseel largely depends on the specific clinical scenarios. As mentioned above, for wound beds, Tisseel may be preferable because it is more retentive and remains in place, helping prevent leakage and maximize therapeutic effect [64]. However, when a flowable delivery system is needed, Tisseel is not ideal because it polymerizes immediately. In such cases, a flowable collagen hydrogel is more suitable (e.g., Bellafill, Suneva Medical, San Diego, CA, USA) Collagen, a major component of the extracellular matrix, has long been used as a scaffold for tissue engineering. Unlike Tisseel, collagen hydrogel takes about 80 s to gel at body temperature, making it more suitable as a flowable hydrogel for injections, such as through a cystoscope or deep tissue arrival. Collagen serves as a viscous, injectable carrier (injectable hydrogel) for PEP [50,74]. Carrier selection should be tailored to the individualized wound and route of administration. Rather than a single optimal option, the ideal scaffold may differ based on tissue type, anatomical location, and clinical needs.

### 4.3. PEP-HA (Including Plated Serum for Skin and Hair)

Hyaluronic acid (HA) is widely used in dermatology, aesthetic medicine, and injectable therapies due to its hydrating and viscoelastic properties. HA has a long history of safe and satisfactory clinical use for local application [76,77,78,80]. HA-based PEP has been used in a rat biceps femoris musculocutaneous flap under ischemia–reperfusion injury. In this model, PEP loaded within an HA hydrogel enabled sustained local exosome retention for up to 72 h in vivo and gradual release over seven days in vitro. PEP-HA treatment achieved reduced serum muscle injury markers, decreased myonecrosis, and modulation of oxidative stress pathways in skeletal muscle, suggesting protection against ischemia-induced oxidative injury. Notably, HA itself appeared to contribute to stabilization of skin perfusion and endothelial function, while the addition of PEP provided muscle-specific protective effects [81]. HA has also been used as a carrier for intra-articular injection of PEP, leveraging its established role as a biocompatible treatment in joint disease. In a rat model of surgically induced osteoarthritis (OA), PEP-HA enabled sustained local release of exosomes following ultrasound-guided intra-articular administration. Treatment with PEP-HA significantly improved functional outcomes, including gait performance and pain-related behaviors, while preserving articular cartilage structure and promoting balanced subchondral bone remodeling, compared to HA alone. Mechanistically, HA-delivered PEP reduced chondrocyte apoptosis and enhanced protective autophagy by modulating the BCL2–Beclin-1 signaling axis, restoring autophagy–apoptosis homeostasis in the joint. These findings indicate that HA functions not only as a topical agent in wound care, but also as an effective carrier for intra-articular injection [38]. A Phase 1b open-label, multicenter clinical trial is currently ongoing to evaluate the safety and exploratory efficacy of intra-articular PEP injection for the treatment of knee OA. The study includes treatment arms evaluating PEP alone and PEP in combination with EUFLEXXA^®^ (Ferring Pharmaceuticals Inc., Parsippany, NJ, USA), an FDA-approved HA, to assess the safety of HA-based delivery in OA patients (https://clinicaltrials.gov/study/NCT06463132?cond=NCT06463132, accessed on 11 November 2025).

**Figure 8:**
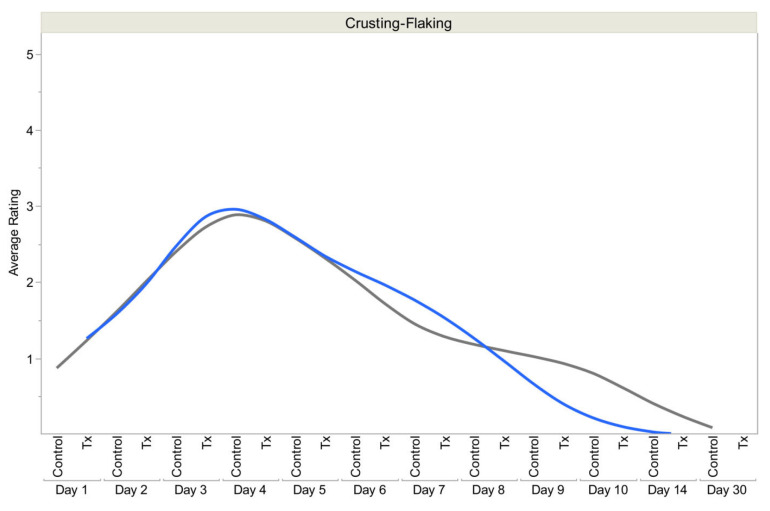
Post-laser treatment use of (Plated)™ serum showing crusting resolved about 5 days sooner in the (Plated)™ group (blue line) than in the control group (black line) with only silicone gel applied post-procedure (*p* < 0.05). (Reprinted from Ref. [77]).

In aesthetic medicine, (Plated)™ serums from Rion Aesthetics (Rochester, MN, USA), which incorporates Human Platelet Extract (HPE) into an HA-based formulation, have demonstrated improvements in skin redness, irritation, inflammation, tone, texture, and smoothness in clinical studies [50,76,77,80]. The (Plated)™ Skin Science system utilizes trillions of bioactive exosomes delivered within an HA matrix for topical use. It can be offered as another use of human platelet-derived exosomes, but only for topical cosmetic applications. In a randomized clinical trial evaluating skin recovery after fractional CO_2_ laser resurfacing, patients treated with topical PEP (Plated)™ serum (Tx group) demonstrated significantly improved healing compared with those receiving standard post-procedure care alone (control group). Standard care included a silicone-based gel (Stratacel^®^, Stratpharma AG, Basel, Switzerland) immediately after the procedure, with routine moisturizing ointment (Vanicream, Pharmaceutical Specialties Inc., Rochester, MN, USA) as needed during the healing period, followed by gentle cleanser and sun protection after re-epithelialization. The Tx group underwent the same laser procedure and standard care regimen, with the addition of topical (Plated)™ serum applied immediately post-procedure and three times daily during healing. Early wound recovery was reported using patient-reported crusting/flaking scores, surveyed daily on a 0–5 ordinal scale, where lower scores indicated reduced scabbing and peeling. The Tx group demonstrated more rapid resolution of crusting/flaking, with significantly lower scores by day 10 compared with the control. Overall aesthetic recovery was further evaluated using the Global Aesthetic Improvement Scale (GAIS), an investigator-assessed measure of cosmetic improvement relative to baseline, which showed superior outcomes in the Tx group (Figure 8) [77].

Together, these data highlight the versatility of PEP across multiple delivery systems, with each formulation tailored to specific therapeutic needs. While fibrin sealant (Tisseel) remains the predominant carrier for wound-bed applications, HA-based systems have demonstrated more value in dermatologic and injectable indications.

## 5. PEP as a Next Generation Delivery System for Chronic Wound Healing

In addition to the inherent and intrinsic regenerative capabilities of PEP, it can also serve as a potential and effective drug delivery system. As mentioned above, PEP represents a next generation delivery platform distinguished by its clinical-grade manufacturing, scalability, and translational readiness, rather than by exosome biology alone. Unlike many experimental exosome preparations, PEP is produced under GMP conditions as a lyophilized, shelf-stable product with consistent particle size, preserved bioactivity, and lot-to-lot reproducibility, enabling reliable clinical translation.

PEP can deliver drugs by using the exosomes as natural nanocarriers to transport various therapeutic agents, such as antibiotics, chemotherapeutics, growth factors and siRNA/mRNA to target areas [10,83]. The lipid bilayer surrounds and protects therapeutic particles and can cross biological barriers, offering a way to strengthen drug biocompatibility, improve drug stability, extend drug half-life and potentially reduce toxicity compared to traditional routes. The massive scaling of manufacturing PEP allows exosomes to become the delivery vehicle for large-scale use for clinical settings. Ongoing research is looking to utilize exosomes to deliver DNA, siRNA, mRNA, and other small molecules within the body. This represents a major future direction for exosome technology, with ongoing studies expected to further expand its regenerative potential. These results will be reported upon completion.

## 6. Conclusions and Future Directions

A key conclusion of this review is that there is no single “most effective” carrier for PEP delivery. Instead, the optimal delivery system depends on the type of tissue injury, anatomical location, and clinical application. For example, fibrin sealant (Tisseel) provides superior local retention and sustained release for wound care and has shown the promising outcomes in ischemic injury, tendon repair, nerve regeneration, and radiated tissue. Collagen-based systems offer a flowable, injectable alternative suitable for deep or minimally invasive delivery, while HA-based formulations are particularly well suited for dermatologic, aesthetic, and topical indications. HA is also an ideal option for intra-articular injections, as it is a natural lubricant in healthy joints. For systemic delivery approaches, such as intravenous administration or nebulization, reconstituted PEP in aqueous solution is sufficient, as no additional delivery system is required.

While PEP administered alone in aqueous solution demonstrated rapid clearance in vivo, this limitation emphasizes the importance of developing controlled delivery systems for PEP and does not diminish PEP’s translational potential. Moreover, future research will continue to prioritize the use of FDA-approved and commercially available materials that are already widely used in clinical practice as carriers for PEP. While other biomaterials may potentially offer advantages for PEP delivery, their development would require unpredictable timelines for regulatory approval and clinical trials and may ultimately not succeed in translation. Accordingly, developing wound healing strategies that can benefit patients as rapidly and reliably as possible is the future for PEP research.

Although this review focuses on treatment for chronic wounds, promising evidence suggests that the therapeutic potential of PEP extends well beyond wound applications. As listed in Table 1, preclinical and early clinical studies have also explored the use of PEP in diverse conditions, such as tendon repair, osteoarthritis, volumetric muscle loss, and cardiac inflammation, demonstrating its broad clinical benefits across multiple tissue types, with shared mechanisms relevant to tissue repair, such as modulation of inflammation, promotion of angiogenesis, and enhancement of cellular migration and remodeling. As PEP continues to advance through clinical development, future research will need to define its role in these non-cutaneous indications and identify tissue-specific delivery strategies and dosing paradigms. These ongoing and future studies position PEP as a regenerative biologic with potential impact across multiple fields of reconstructive and regenerative medicine.

## Figures and Tables

**Table 1:** Currently Tested Delivery Strategies for PEP in Regenerative Medicine.

Delivery Platform	Healing Model	Key Outcomes	Ref.
Aqueous solution (PEP-only)			
PEP + PBS	Canine tenocytes in vitro	Effective cell uptake of PEP, ↑ proliferation/migration; ↑ SCX, COL1A1, COL3A1, TNMD, DCN, MKX; reduced dexamethasone-induced apoptosis	[59]
PEP + sterile water	Human epithelial adenocarcinoma cells (representing the glandular and luminal epithelium), human stromal cells and menstrual blood-derived stem cells in vitro to represent endometrium	↑ endometrial cell proliferation and wound healing capacity in vitro, PEP absorbed by cells	[66]
PEP + PBS	C28/I2 cells and chondrocytes-derived from OA patients in vitro	Fast cell uptake, ↑ cellular proliferation and migration, ↓ apoptosis by reducing CASP3/7/9 and BAX.	[65]
PEP + PBS for intraperitoneal injection	Mice myocarditis model in vivo	↓ inflammatory cells and proinflammatory and profibrotic markers, Immunoregulatory	[63]
PEP + PBS for nebulization, intravenous (IV) injection and pulmonary artery balloon catheter	Porcine lung biodistribution model in vivo	Global delivery by nebulization or IV, targeted region delivery by catheter	[69]
PEP + distilled water for nebulization	Mice emphysema model in vivo	Effectively delivered into injured lung, ↓ smoke-induced apoptotic cell death and emphysema, ↓ oxidative lung injury and inflammation	[60]
Fibrin sealant *			
PEP + Tisseel	Rat sciatic nerve autograft repair model in vivo	↑ GAP43 and S100b expression, ↑ axon diameter and maturation, ↑ motor functional recovery	[70]
PEP + Tisseel	Rat rotator cuff repair model in vivo	Improved tendon-bone integration, ↑ tendon Col1/col3/SCX/Tnmd/TNC/DCN/IGF expression, ↑ healing speed and strength	[68]
PEP + Tisseel	Rabbit ear ischemic wound in vivo	↑ angiogenesis, mature skin function, ↑ regenerative pathways including regulating downstream mediators of TGF-β (RHOA, SMAD2, TAK1 and RAS)	[17]
PEP + Tisseel	Canine tendon repair model ex vivo	↓ inflammation, gap formation, ↓ tendon healing by enhancing endogenous tenocytes and type II collagen	[67]
PEP + Tisseel	Rat latissimus dorsi defect model in vivo	↑ skeletal muscle regeneration and polarization of local macrophages towards the regenerative M2 phenotype, ↓ inflammation and fatty infiltrate	[50]
PEP + Tisseel	Rat volumetric muscle loss model in vivo	↑ skeletal muscle regeneration, ↑ functional recovery and more cellularity compared to control	[62]
PEP + Tisseel	Rat sciatic nerve allograft repair model in vivo	Taken up by Schwann cells fast, ↑ Schwann cell viability and migration for healing, ↑ motor functional recovery	[71]
PEP + Tisseel	Nonhealing scalp wound after chemoradiation in human	Wounds healed successfully despite resistance to conventional wound care with PEP remaining securely in place	[64]
PEP + Tisseel	Rat rotator cuff injury model in vivo	↑ collagen type 1 & 3 and TGF-β, accelerated tendon-bone healing with improved biomechanical strength and recovered gait	[72]
Collagen *			
PEP + Type-1 collagen scaffold	Rabbit Achilles tendon repair model in vivo	↓ external adhesions macroscopically and microscopically	[73]
PEP + Type-1 collagen hydrogel injection	Porcine vaginal mesh exposure model in vivo	↑ regenerated epithelial tissue over mesh, ↓ inflammation and fibrosis, ↑ capillary density,	[74,75]
PEP bipotentiated type-1 collagen hydrogel	Porcine stress urinary incontinence (skeletal muscle injury) model in vivo	Restored muscle function, ↑ new myofibers and M2 macrophage polarization	[50]
PEP + Collagen mixture through a sterile polyurethane film	Nonhealing scalp wound after chemoradiation in human	Wounds healed successfully despite resistance to conventional wound care with PEP leaking	[64]
Hyaluronic acid			
Topical HA-based HPE serum	Twice daily facial skin application in human	Improvements in general skin health, ↓ redness, wrinkles, and melanin production	[76]
Topical HA-based HPE serum with methyl	Post-laser skin application in human	Cooling function, ↓ post-procedure skin trauma and recovery time	[77]
Topical HA-based HPE serum	Twice daily on face wrinkles in human	↓ cellular senescence markers p16INK4a and p21CIP1/WAF1, ↓ senescence-associated telomere damage, ↓ inflammation, ↑ collagen and elastin-related genes and fibers	[78,79]
Topical HA-based HPE hair serum	Daily hair-scalp application in human	↑ hair density, size, volume fullness, scalp coverage, and overall health of the hair	[80]
PEP + HA	Rat ischemia–reperfusion injury model in a musculocutaneous flap	↓ serum creatinine kinase and myonecrosis, modulation of iNOS	[81]
Intra-articular Injection of PEP + PBS + Hylan G-F 20 *	Rat osteoarthritis model	↓ BCL2 expression and chondrocyte apoptosis, ↑ proteins LC3 and Beclin-1	[38]

PEP: purified exosome product, ↑: increase, ↓: decrease, OA: osteoarthritis, PBS: phosphate-buffered saline, HA: hyaluronic acid, HPE: human platelet extract. * PEP was reconstituted in PBS according to manufacturer instructions for the carrier systems.

## Data Availability

No new data were created or analyzed in this study. Data sharing is not applicable to this article.
